# Effect of Retreatment Protocols on the Push‐Out Bond Strength of Hydraulic Calcium Silicate Cement in Open‐Apex Teeth

**DOI:** 10.1155/tswj/8891215

**Published:** 2026-06-19

**Authors:** Faraz Seyedforootan, Noushin Shokouhinejad, Fatemeh Hamidzadeh

**Affiliations:** ^1^ School of Dentistry, Tehran University of Medical Sciences, Tehran, Iran, tums.ac.ir; ^2^ Department of Endodontics, School of Dentistry, Tehran University of Medical Sciences, Tehran, Iran, tums.ac.ir

**Keywords:** calcium silicate–based cement, mineral trioxide aggregate, nonsurgical root canal retreatment, open apex, push-out bond strength, solvent, ultrasonic

## Abstract

**Objectives:**

During retreatment, complete removal of previous obturation materials is essential, as remnants may compromise adhesion. This study evaluated the effect of four retreatment protocols on the push‐out bond strength of RetroMTA (BioMTA, Seoul, Korea), a fast‐setting calcium silicate–based material, used as an apical barrier in teeth with open apices that had been previously obturated with gutta‐percha and AH26 sealer.

**Materials and Methods:**

Sixty extracted single‐rooted human teeth were instrumented and obturated using lateral condensation and then stored in phosphate‐buffered saline (PBS) at 37°C for 4 weeks. Specimens were randomly assigned to four groups (*n* = 15): mechanical retreatment, mechanical retreatment with passive ultrasonic irrigation (PUI), chloroform‐assisted retreatment, and chloroform‐assisted retreatment with PUI. After retreatment, the apical 5 mm of each canal was obturated with RetroMTA and stored for another 4 weeks. Push‐out bond strength was measured using a universal testing machine. Data were analyzed using two‐way ANOVA. The significance level was set at *α* = 0.05.

**Results:**

Chloroform use did not significantly affect the push‐out bond strength of RetroMTA (*p* = 0.349). In contrast, PUI significantly increased bond strength regardless of solvent use (*p* < 0.001). No significant interaction was observed between chloroform and PUI.

**Conclusion:**

Within the limitations of this ex vivo study, PUI significantly enhanced the bond strength of RetroMTA to root dentin, whereas chloroform provided no additional benefit.

## 1. Introduction

Root development and apical closure in permanent teeth generally continue for approximately 3–4 years after eruption. During this developmental period, deep carious lesions or traumatic injuries may result in pulpal necrosis, leading to disruption of normal root maturation. Consequently, immature permanent teeth with necrotic pulps are characterized by thin dentinal walls, open or irregular apices, and an absence of apical constriction, all of which render endodontic treatment more complex and technically demanding [1, 2].

Hydraulic calcium silicate–based cements (HCSCs), including mineral trioxide aggregate (MTA), are widely used in a variety of clinical applications, such as root‐end filling, perforation repair, pulp capping, apexogenesis, and the management of immature and mature teeth with necrotic pulps and open apices [3]. The introduction of HCSCs has enabled more predictable and time‐efficient treatment of immature teeth, thereby overcoming several limitations associated with prolonged calcium hydroxide apexification procedures [4].

RetroMTA, a fast‐setting HCSC containing calcium carbonate, aluminum oxide, silicon oxide, and a calcium–zirconia complex, uses zirconium oxide instead of bismuth oxide to reduce tooth discoloration and achieve quicker setting [5, 6].

In teeth with necrotic open apices that have previously undergone root canal obturation with gutta‐percha and sealer but subsequently failed, nonsurgical retreatment followed by placement of an HCSC apical barrier is often indicated. The objectives of retreatment include removing the existing root filling materials, effectively disinfecting the root canal system, and re‐establishing conditions favorable for periapical healing prior to reobturation. Thorough removal of the previous obturation is essential, as residual filling materials may hinder canal disinfection and adversely affect the adaptation and sealing ability of the subsequent filling material [7].

Despite the availability of numerous retreatment techniques, no consensus has been reached regarding the most effective method for removing obturation material. Gutta‐percha and sealers may be removed mechanically using hand or rotary instruments, with or without the use of chemical solvents [8]. Chloroform remains one of the most commonly used solvents due to its rapid and effective gutta‐percha softening capability [9]. To achieve complete debridement of the root canal system, adjunctive approaches are frequently recommended; including the use of sonic and ultrasonic instruments can be mentioned [10]. Passive ultrasonic irrigation (PUI) has been shown to enhance irrigant efficacy through cavitation and acoustic streaming, thereby facilitating smear layer and debris removal in inaccessible areas. Compared with syringe irrigation, PUI improves the elimination of microorganisms and residual filling materials [11].

Numerous studies have demonstrated that the push‐out bond strength of HCSCs is influenced by multiple factors, including the type and concentration of irrigants, intracanal medicaments, environmental conditions such as pH and moisture, and dentin substrate characteristics [12–14].

Solvents used during retreatment to facilitate the removal of gutta‐percha and sealer may also affect the bond strength of the reobturation materials. Several studies have investigated the effect of various solvents, including chloroform, on the push‐out bond strength of different types of sealers. Some of these studies have reported a reduction in sealer bond strength [15], whereas others have found no significant difference regardless of solvent use [16]. Although the existing literature extensively addresses the removal of gutta‐percha and associated sealers, a significant knowledge gap remains regarding the effects of retreatment protocols on the adhesion of HCSCs to root canal dentin, particularly in immature teeth with open apices, where HCSCs serve as a secondary obturation material. Therefore, this study is aimed at comparing different retreatment strategies, including the use of chloroform and PUI, on push‐out bond strength of RetroMTA when used as secondary apical barrier materials.

## 2. Materials and Methods

This ex vivo study was approved by the Research Ethics Committee of Tehran University of Medical Sciences (IR.TUMS.DENTISTRY.REC.1402.117).

### 2.1. Sample Size Calculation

Sample size calculation was performed using the PASS 11 software (NCSS, LLC, Kaysville, Utah, United States). Based on a previous study [17], using the fixed‐effect analysis of variance (ANOVA) power analysis module, a sample size of 10 per subgroup was required to detect a significant difference in push‐out bond strength between two different retreatment methods (effect size = 0.462) and two different irrigation techniques (effect size = 0.769) with *α* = 0.05, yielding statistical powers of 0.81 and 0.98, respectively. Considering potential dropouts during the study, 15 samples were assigned to each subgroup.

### 2.2. Specimen Preparation

The specimens were selected from teeth extracted due to severe coronal caries, periodontal diseases, or orthodontic indications. Sixty single‐rooted human teeth with closed apices were included. Standardized periapical radiographs were obtained in both buccolingual and mesiodistal directions for all specimens. Teeth presenting with multiple canals, calcification, root resorption, cracks, caries, or existing restorations were excluded. The selected teeth were disinfected in 0.5% chloramine T solution for 1 week and stored in normal saline until use.

**Figure 1:**
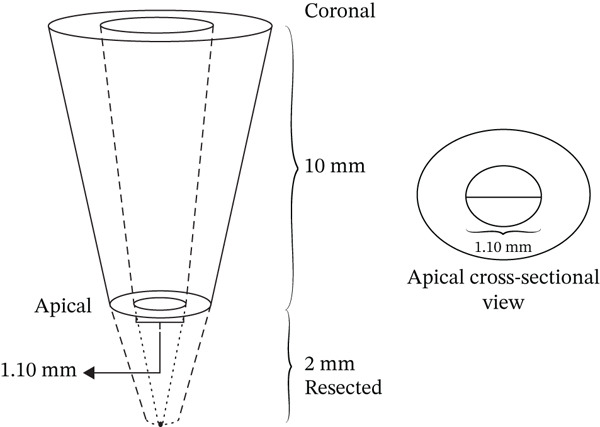
Schematic view of the simulated open‐apex specimen.

To simulate open apex conditions, a 2‐mm apical resection was performed. Each root was then shortened to a final length of 10 mm, measured from the buccal cementoenamel junction (CEJ), using a high‐speed handpiece and a diamond bur. Canal preparation was performed using the Denco Blue Super Files III rotary system (Denco, Shenzhen, China) up to size F3. Gates–Glidden drills (Sizes #1–#4) were advanced sequentially beyond the resected apex, thereby creating a wide apical opening comparable to that of immature permanent teeth with open apices (Figure 1). During instrumentation, irrigation was performed with 2 mL of 2.5% sodium hypochlorite between each file or drill. Smear layer removal was then carried out using the following irrigation sequence: 5 mL of 2.5% sodium hypochlorite for 1 min, followed by 5 mL of normal saline, 5 mL of 17% EDTA for 1 min, and a final rinse with 5 mL of normal saline. The canals were then dried using paper points (Meta Biomed, Meta, South Korea). Canals were obturated with gutta‐percha (Meta Biomed, Meta, South Korea) and AH26 sealer (Dentsply, Konstanz, Germany) using the lateral condensation technique. The coronal access was sealed with a temporary filling material. To ensure complete setting of the sealer, the teeth were then immersed in PBS at 37°C for 4 weeks.

Subsequently, the specimens were randomly assigned to four groups (*n* = 15 per group) according to the retreatment protocols described below.

#### 2.2.1. Group 1: Retreatment Without the Use of Chloroform or PUI (Chloroform−/PUI−)

The coronal 3 mm of the root canal filling was removed using a Gates–Glidden drill. Gutta‐percha was subsequently removed with D1–D3 files of the Denco D Files retreatment system (Denco, Shenzhen, China) according to the manufacturer′s instructions, supplemented with Hedström files (Mani, Japan) to ensure complete removal of the obturation material. During instrumentation, irrigation was performed with 2 mL of 2.5% sodium hypochlorite between each instrument. Final irrigation consisted of 5 mL of 2.5% sodium hypochlorite for 1 min, followed by normal saline, 5 mL of 17% EDTA for 1 min, and a final rinse with 5 mL of normal saline.

#### 2.2.2. Group 2: Retreatment Without the Use of Chloroform and With Supplementary Cleaning Using PUI (Chloroform−/PUI+)

The mechanical retreatment protocol described for Group 1 was followed. During the final irrigation phase, 2.5% NaOCl and 17% EDTA were introduced into the canal and ultrasonically activated for 30 s in continuous mode using a gold ultrasonic tip (length: 18 mm; size: 20/2%) attached to an ultrasonic activator operating at a frequency of 45 kHz (Ultra X, Eighteeth, Changzhou Sifary Medical Technology Co., Ltd., Changzhou, Jiangsu, China). The ultrasonic tip was inserted 8 mm into the canal during activation.

#### 2.2.3. Group 3: Retreatment Using Chloroform Without PUI (Chloroform+/PUI−)

The coronal 3 mm of the root canal filling was removed using a Gates–Glidden drill. The prepared coronal reservoir was then filled with approximately 0.4 mL of chloroform and allowed to stand for 1 min before proceeding with the remaining retreatment steps. Chloroform was applied only once at the beginning of the retreatment procedure and was not refreshed during apical instrumentation. Mechanical removal of the obturation material was then carried out using the same instrumentation sequence described for Group 1. The irrigation protocol and final rinse regimen were identical to those used in Group 1.

#### 2.2.4. Group 4: Retreatment Using Chloroform and PUI (Chloroform+/PUI+)

Chloroform was applied as described for Group 3, and supplementary cleaning with passive ultrasonic activation of the final irrigants was carried out as described for Group 2.

The total volume of irrigant and the duration of irrigation were standardized across all groups. In groups receiving PUI, the 30‐s PUI procedure utilized the same total volume of irrigant as the 30‐s conventional syringe irrigation applied in the control groups.

Following retreatment, RetroMTA cement (BioMTA, Seoul, Korea) was prepared according to the manufacturer′s instructions and compacted into the apical 5 mm of each root canal to create an apical barrier. The roots were stabilized in a sponge to facilitate handling during material placement. A moist paper point was placed coronally, and the coronal portion of the canal was temporarily sealed. All specimens were stored in PBS at 37°C for 4 weeks to allow complete setting of the cement prior to push‐out bond strength testing.

### 2.3. Push‐Out Bond Strength Test

After a 4‐week storage period, specimens were embedded in polyester resin. From the apical 5 mm of each root, two root slices were sectioned using a diamond disc (Mecatome, Presi, France) with a thickness of 1.00 ± 0.05 mm. The thickness of each slice was verified using a digital caliper. The root slices were examined under ×15 magnification, and images were captured from the apical and coronal sides of the root slices to measure the perimeter of the filling RetroMTA cement. After that, the filling material was loaded with a 1‐mm diameter cylindrical stainless‐steel plunger. Loading was performed using a universal testing machine (Z050, Zwick/Roell, Ulm, Germany) at a speed of 0.5 mm/min in the apical coronal direction until bond failure occurred. The maximum load applied to the filling material before debonding was recorded in newtons. The push‐out bond strength value was calculated in megapascals (MPa) by dividing the load at failure in newtons (N) by the adhesion area, determined from the formula *P* × *h*, where *P* is the mean perimeter of the filling material at the apical and coronal sides and *h* is the thickness of the root slice in millimeters.

**Figure 2:**
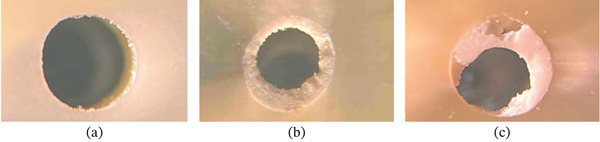
Various failure modes: (a) adhesive, (b) cohesive, and (c) mixed.

After the push‐out testing, the root slices were examined under ×15 magnification to determine the failure mode: adhesive (at the cement/dentin interface), cohesive (within the cement), or mixed failure (both adhesive and cohesive failure modes) (Figure 2).

### 2.4. Data Analyses

Statistical analyses were performed using SPSS Statistics software (Version 26; IBM Corp., Chicago, IL, United States). The normal distribution of push‐out bond strength data was assessed using the one‐sample Kolmogorov–Smirnov test. A two‐way ANOVA was conducted to evaluate the main effects of chloroform application (yes/no) and PUI (yes/no), as well as their interaction, on the push‐out bond strength of RetroMTA. The level of statistical significance was set at *p* < 0.05.

## 3. Results

**Figure 3:**
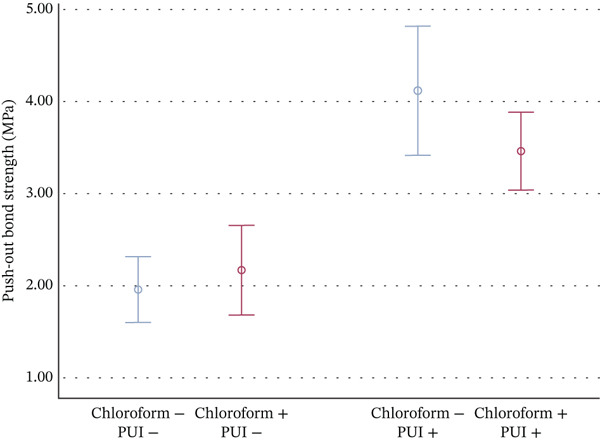
Mean and standard deviation of the push‐out bond strength (MPa) of RetroMTA in different groups of study.

The mean push‐out bond strength values are shown in Figure 3 and Table 1.

**Table 1:** Mean push‐out bond strengths (±SD) and failure modes for the experimental groups.

Group (N = 15)	Mean ± SD (MPa)	Failure mode (%)		
		Adhesive	Cohesive	Mixed
Chloroform−/PUI−	1.96 ± 0.65	20%	6.6%	73.3%
Chloroform−/PUI+	4.12 ± 1.26	6.6%	33.3%	60%
Chloroform+/PUI−	2.17 ± 0.88	20%	6.6%	73.3%
Chloroform+/PUI+	3.46 ± 0.76	20%	0%	80%

No statistically significant interaction was observed between chloroform application and PUI (*p* = 0.073). The use of chloroform alone did not significantly influence the push‐out bond strength of RetroMTA (*p* = 0.349). In contrast, the application of PUI resulted in significantly higher bond strength values, regardless of chloroform use (*p* < 0.001). Accordingly, RetroMTA exhibited significantly greater bond strength in all groups in which PUI was applied. The bond failure patterns for each study group are presented in Table 1.

Inspection of the root‐filled discs under magnification revealed that the bond failure in all the groups was mostly mixed. Failure modes were evaluated descriptively to provide an observational comparison among the groups, and no statistical analysis was performed for these data.

## 4. Discussion

This study investigated the effect of chloroform use and adjunctive ultrasonic irrigation during retreatment of root canals previously obturated with gutta‐percha and AH26 sealer on the push‐out bond strength of RetroMTA cement to root dentin after refilling with RetroMTA. The push‐out test, a widely accepted method for evaluating resistance to dislodgement and assessing the bond strength between root canal filling materials and dentin, was employed [18].

To partially simulate clinical conditions, the specimens were stored in PBS following either primary endodontic treatment or retreatment. PBS is considered a synthetic tissue fluid due to its ionic composition, which closely resembles that of physiological fluids. Previous studies have demonstrated that exposure of calcium silicate–based materials to phosphate‐containing solutions promotes apatite crystal formation at the material–dentin interface, which may enhance interfacial bonding and improve material adhesion [19, 20].

The present study employed PUI during retreatment and in the final phase of root canal preparation. Previous studies have demonstrated that PUI enhances the efficacy of irrigants in cleaning the root canal system during retreatment procedures [21, 22]. This approach addresses the persistence of debris and microorganisms within the complex anatomy of the root canal system, thereby improving overall disinfection and cleanliness [23, 24]. The findings of this study revealed that the push‐out bond strength of RetroMTA was significantly higher in the groups in which ultrasonic activation was applied during retreatment. This outcome may be attributed to the more effective removal of gutta‐percha and sealer remnants from the canal walls when PUI is used, resulting in cleaner dentin surfaces and closer direct contact between RetroMTA and root dentin. In contrast, residual gutta‐percha and sealer may hinder intimate contact between calcium silicate–based cements and dentin, thereby compromising adhesion. Consistent with the present results, several studies have reported that PUI improves the removal of gutta‐percha and epoxy resin–based sealers during retreatment procedures [22, 25, 26]. In addition to the improved elimination of obturation remnants, ultrasonic activation of irrigants may also increase the effectiveness of irrigation solutions in removing both organic and inorganic components of the smear layer [27]. This effect may further promote closer adaptation and improved bonding of calcium silicate–based cements to root dentin.

In the present study, chloroform was used as a gutta‐percha solvent during retreatment. Chloroform is an organic solvent with high efficacy in dissolving gutta‐percha and remains one of the most widely used agents for this purpose [9]. The results of this study demonstrated that chloroform application did not significantly influence the push‐out bond strength of RetroMTA to root dentin. This is consistent with the study by Bayram et al. [28] who reported that the bond strength of white mineral trioxide aggregate (WMTA) was not adversely affected following 5 min of chloroform exposure, although reductions were observed for other calcium silicate–based materials, including capsule‐form MTA (CMTA) and Biodentine. However, methodological differences should be noted between studies. In the present investigation, chloroform was applied during the initial phase of retreatment, whereas Bayram et al. [28] exposed dentin surfaces directly to chloroform for 5 min prior to placement of calcium silicate–based cements. Furthermore, the potential effect of chloroform on the bond strength of some HCSCs may be related to its influence on dentin surface characteristics during retreatment. Although chloroform facilitates the dissolution of gutta‐percha and sealer remnants, it may also alter the dentin substrate by affecting the organic matrix and surface morphology. Such changes could modify dentin permeability and smear layer characteristics, potentially influencing the interaction between dentin and HCSCs. Since the adhesion of these materials is associated with micromechanical interlocking and biomineralization processes at the dentin–material interface, alterations in the dentin surface may affect ion exchange and the formation of calcium–phosphate deposits. Therefore, differences reported among studies may be attributed to variations in solvent exposure protocols, irrigation procedures, and the physicochemical properties of the materials evaluated.

The choice of solvents during retreatment may influence bond strength by altering dentin surface characteristics and their interaction with the filling material. Variations in reported outcomes may be attributed to differences in obturation and sealer type, the calcium silicate cement used, the type of solvent, and the duration and mode of solvent application. For instance, prolonged dentin–chloroform contact (5 min) has been shown to significantly reduce the push‐out bond strength of Sealapex, AH Plus, and MTA Fillapex, whereas a shorter exposure time (2 min) had no significant effect [14]. Solvents can facilitate the dissolution of canal filling materials during retreatment; however, they may also generate a softened gutta‐percha and sealer layer that adheres to canal walls, thereby complicating effective debridement. In the present study, the bond strength of RetroMTA in canals retreated with chloroform did not differ significantly from that observed in canals cleaned mechanically without solvent use, suggesting that chloroform application did not adversely affect the RetroMTA–dentin bond. Ultrasonic activation appeared to enhance the removal of gutta‐percha and AH26 remnants, even when softened by chloroform. Therefore, the use of PUI may counteract potential adverse effects of solvent residues by improving dentin surface cleanliness and creating more favorable conditions for RetroMTA bonding.

Mixed failure was the predominant mode observed across all experimental groups, reflecting variations in dentin surface conditions following retreatment. Residual filling materials or sealer remnants may interfere with adhesion, leading to localized separation of RetroMTA from root dentin. Notably, the chloroform−/PUI+ group demonstrated a higher incidence of cohesive failure compared with the other groups. Provided that the cement itself does not exhibit inherent structural weakness, cohesive failure indicates that the bond strength at the cement–dentin interface exceeds the internal strength of the material, resulting in fracture within the bulk of the cement rather than at the interface. Although mixed failures were dominant, the overall distribution still provides useful insight into interfacial behavior. The slightly higher rate of cohesive failures in the chloroform−/PUI+ group suggests a more robust cement–dentin interface under this condition, supporting the positive effect of ultrasonic activation on RetroMTA stability after retreatment. In the study by Bayram et al. [28], WMTA and CMTA predominantly exhibited adhesive failure modes, whereas Biodentine showed mainly mixed failures. These differences among studies may be attributed to variations in the physicochemical properties of the calcium silicate–based cements evaluated, as well as variations in experimental design. Specifically, in Bayram et al.′s study [28], the lumens of root discs were directly exposed to chloroform for 5 min prior to placement of calcium silicate cements, whereas in the present study, chloroform was applied only during the initial phase of gutta‐percha removal. Previous investigations have also reported inconsistent failure patterns for calcium silicate–based cements in bond strength testing [29, 30].

Our results showed that the use of PUI significantly increased the push‐out bond strength of RetroMTA to root dentin in previously filled canals, irrespective of chloroform application. From a clinical standpoint, these findings suggest that ultrasonic activation of irrigants during retreatment can be recommended to enhance the interfacial resistance and potential long‐term stability of RetroMTA as a root canal filling material. Furthermore, the use of chloroform for gutta‐percha removal did not significantly reduce the bond strength of RetroMTA, which may reassure clinicians who routinely employ chloroform in retreatment procedures. It should be acknowledged that the displacement resistance and failure modes observed in the present study cannot be directly compared with those reported in studies in which calcium silicate–based cements were used as primary root canal filling materials. Moreover, the findings are specific to RetroMTA and should not be generalized to other calcium silicate–based cements that differ in composition, setting kinetics, or interfacial behavior. The results are also limited to the retreatment protocols, solvents, irrigation regimens, and exposure times evaluated in this study. Although the ex vivo design allowed standardized conditions, some limitations should be acknowledged. This model cannot fully reproduce the complex biological environment of retreatment in immature teeth with open apices. In particular, the absence of periodontal ligament and periapical tissues may influence stress distribution and material behavior. In addition, the push‐out test applies a simplified unidirectional load, whereas clinical conditions involve multidirectional and cyclic forces. Therefore, the results should be interpreted with caution, and further in vivo and clinical studies are recommended to confirm their clinical relevance.

## 5. Conclusion

Within the limitations of this ex vivo study, the bond strength of RetroMTA was significantly higher in groups in which PUI was applied during the retreatment of root canals previously obturated with gutta‐percha and AH26 sealer, regardless of chloroform use. From a clinical perspective, the application of PUI during retreatment may enhance the bonding performance of calcium silicate–based materials when canal refilling is required.

## Funding

This study was funded and supported by Tehran University of Medical Sciences (Grant no.71903)

## Conflicts of Interest

The authors declare no conflicts of interest.

## Data Availability

The data that support the findings of this study are available on request from the corresponding author. The data are not publicly available due to privacy or ethical restrictions.
